# Red flags and adjusted suspicion index for distinguishing hereditary transthyretin amyloid polyneuropathy from idiopathic axonal polyneuropathy

**DOI:** 10.1007/s10072-023-06859-w

**Published:** 2023-06-02

**Authors:** Janna K. Warendorf, Gerjan M. van der Star, Dennis Dooijes, Nicolette C. Notermans, Alexander F. J. E. Vrancken

**Affiliations:** 1 Department of Neurology, UMC Utrecht Brain Center, University Medical Center Utrecht, 3584 CX Utrecht, The Netherlands; 2 Department of Genetics, University Medical Center Utrecht, 3584 CX Utrecht, The Netherlands

**Keywords:** Polyneuropathy, Neuropathy, Genetics, Amyloidosis, ATTRv-PN, Transthyretin, ATTR

## Abstract

**Background:**

Early diagnosis of hereditary ATTR polyneuropathy (ATTRv-PN) is important since treatment options have become available, which are most effective early in the disease course. ATTRv-PN is likely underdiagnosed as patients might be misdiagnosed with idiopathic polyneuropathy. It is uncertain if it is useful to test for TTR gene mutations in patients with a typical presentation for chronic idiopathic axonal polyneuropathy (CIAP) and which are the distinguishing clinical features.

**Methods:**

We carried out a retrospective cohort study to assess the yield of TTR gene sequencing in patients with polyneuropathy and assessed if the identified patients with ATTRv-PN had a clinical presentation typical of CIAP. Additionally, we assessed which clinical features, including previously defined red flag symptoms, can differentiate between patients with CIAP and ATTRv-PN and assessed the performance of the TTR suspicion index.

**Results:**

Out of 338 patients with polyneuropathy, 10 patients had a pathogenic TTR gene mutation (all p.Val50Met) and none had a clinical presentation typical of CIAP. Patients with ATTRv-PN more often had bilateral CTS, motor involvement of arms, cardiac involvement, family history suggestive of hATTRv, and autonomic symptoms than patients with CIAP. All patients with ATTRv-PN as well as 70% of patients with CIAP fulfilled the suspicion index.

**Conclusion:**

Routine TTR gene sequencing in patients with a typical presentation for CIAP is not useful. However, red flag symptoms can differentiate patients with ATTRv-PN from patients with CIAP. We propose an adjusted version of the TTR suspicion index to increase diagnostic yield.

**Supplementary Information:**

The online version contains supplementary material available at 10.1007/s10072-023-06859-w.

## Introduction

Hereditary transthyretin amyloidosis (hATTRv) is an autosomal dominant multisystem disorder as a result from misfolding of the destabilized tetrameric transthyretin (TTR) protein. Aggregations of misfolded TTR protein can deposit in multiple organs, leading mainly to peripheral neuropathy (ATTRv-PN) and/or cardiomyopathy. Untreated hATTRv ultimately leads to death for most patients within 10–15 years of disease onset [1]. Stabilizing molecules and gene-silencing therapies have become available for ATTRv-PN, which are most successful in the early stage of the disease [2]. It is therefore of increasing importance to prevent diagnostic delays. Unfortunately, hATTRv is likely to be an underdiagnosed and diagnostic delay is common due to phenotypic heterogeneity, especially in patients with late onset, without a family history and in non-endemic regions [3, 4].

In a substantial proportion of patients with polyneuropathy, no underlying etiology can be established after extensive diagnostic work-up [5, 6]. A strategy to identify ATTRv-PN early is routine genetic testing in patients with such apparently idiopathic polyneuropathy. However, it is not known in which subset of patients this is useful. There might be patients with ATTRv-PN presenting with a typical slowly progressive axonal polyneuropathy who are misdiagnosed as chronic idiopathic axonal polyneuropathy (CIAP). Results from previous studies on the yield of genetic testing for hATTRv in idiopathic (poly)neuropathy have been conflicting [7–9].

Several clinical characteristics such as autonomic symptoms, carpal tunnel syndrome (CTS), and cardiomyopathy have been described to be indicative of ATTRv-PN [3, 10, 11]. Most of these “red flags” were defined in patient cohorts in endemic regions. It is not known if these characteristics are applicable to distinguish patients with ATTRv-PN from those without a mutation in non-endemic regions, especially in the early stage of the disease.

Therefore, we investigated the yield of TTR gene sequencing in a Dutch population of patients with polyneuropathy, assessed if the identified patients with ATTRv-PN could have been misdiagnosed as CIAP, and which are potentially the most outstanding and differentiating clinical features.

## Methods

### Yield of genetic testing

Between January 2009 and January 2020, there were 338 patients with chronic polyneuropathy who underwent sequencing of the TTR gene as part of their diagnostic work-up at our hospital, a tertiary medical center in the Netherlands with special expertise in polyneuropathy. The choice to carry out genetic testing was made by the clinician who evaluated the patient and was carried out with a low threshold. We assessed the yield of genetic testing in these patients, re-evaluated the clinical data of the identified patients with ATTRv-PN, and assessed if they could have been misdiagnosed as CIAP (i.e., if they had a clinical presentation typical for CIAP).

A typical presentation for CIAP was defined as follows:

- age at symptom onset > 40 years old,
- length-dependent symptoms (numbness, paraesthesia, pain, or weakness) with distal sensory or sensorimotor signs and/or decreased or absent reflexes upon neurological examination,
- axonal polyneuropathy,
- slow or no progression over at least 6 months,
- no first-degree family members with idiopathic or (suspected) hereditary polyneuropathy, and
- no known identifiable cause of polyneuropathy present after extensive work-up as previously described [12].

### Clinical phenotype

We compared clinical features between patients with CIAP and patients with ATTRv-PN at their first presentation. To include as many patients with ATTRv-PN as possible for this comparison, we also identified all those patients who were referred by other hospitals or the genetics department to our specialized amyloidosis outpatient clinic with an established diagnosis of hATTRv. Patients thus identified were included in addition to those in the yield of the genetic testing group. To allow proper comparisons between CIAP and ATTRv-PN, patients with ATTRv-PN and an additional etiology for polyneuropathy were excluded, as this could have influenced the clinical phenotype.

Clinical data were extracted from the electronic patient files, which included age at onset of symptoms, age at genetic testing, sex, duration of symptoms at TTR testing, family history, medical history, symptoms and localization of symptoms at onset and first presentation, progression rate of symptoms (in days, weeks, months or years), findings upon neurological examination, and results of additional investigations [13].

We compared the presence of previously described red flags for ATTRv-PN, including family history suggestive of hATTRv (polyneuropathy, cardiomyopathy, and/or CTS), cardiac signs suggestive of hATTRv (e.g., peripheral edema, exertional dyspnoea, cardiac arrhythmias), fast progression of symptoms (in weeks or months), neuropathic pain, CTS, walking disability within 5 years of disease onset (i.e., needing a walking aid), and autonomic symptoms, including sicca, palpitations, urinary urgency or incontinence, (alternating) constipation or diarrhea, orthostatic hypotension, sexual dysfunction, early satiety or vomiting, and altered sweating pattern (hypo- or hyperhidrosis) [3, 11]. Additionally, we assessed if there was decreased strength and motor involvement of the upper limbs, as ATTRv-PN patients have been shown to have pronounced decreased strength and more frequent involvement of arms [11].

We also tested how well the suspicion index for diagnosis of ATTRv-PN performed in our study population [10]. This index advises to test for hATTRv in patients with an idiopathic progressive polyneuropathy when 1 or more of the following items are present: dysautonomia (defined as altered defecation pattern, orthostatic hypotension, urinary symptoms, or erectile dysfunction), family history (suggestive) of hATTRv, bilateral CTS, early gait disorder (i.e., needing a walking aid within 5 years of onset of polyneuropathy), unexplained weight loss > 5 kg, cardiac hypertrophy or arrhythmia, vitreous opacities, or renal dysfunction.

To assess whether there was selection bias for genetic testing, we compared the characteristics mentioned above between the patients with CIAP who underwent TTR gene sequencing and patients with CIAP who did not undergo sequencing presenting at our outpatient clinic in the same time period.

### Statistical analysis

All statistical analyses were performed in R-studio (version 1.1.456). Results were considered statistically significant when the *P*-value was less than 0.05. Dichotomous participant characteristics were compared with either the χ2 or Fisher’s exact test. Student’s *t*-test or Mann–Whitney U test was used for continuous variables.

Ethical approval was waived by the local Ethics Committee of the University Medical Center Utrecht in view of the retrospective nature of the study, and all the procedures being performed were part of routine care.

## Results

Within the study period, 338 patients with chronic polyneuropathy underwent TTR gene sequencing, of which 92 patients were ultimately diagnosed with CIAP. Additionally, within the same time period, 162 patients with CIAP were seen at our outpatient clinic who did not undergo TTR gene sequencing.

### Yield of TTR gene sequencing

Table 1 shows the characteristics of all 338 patients with chronic polyneuropathy who were genetically tested. There were 10 patients with the heterozygous pathogenic c.148G > A (p.Val50Met) variant (this variant is also known as p.Val30Met in old nomenclature), and these ten patients were diagnosed with ATTRv-PN. Subsequently, there were 236 tested patients who had a known underlying cause for polyneuropathy other than ATTRv-PN, and 92 patients who were ultimately diagnosed with CIAP.

**Table 1:** Characteristics of tested patients with polyneuropathy

	TTR mutation, *n* = 10	No TTR mutation, *n* = 328	*P* value
Man/women n (%)	9/1 (90.0/10.0)	218/110 (66.4/33.6)	0.17^5^
Age at TTR testing, mean (SD)	68.2 (5.6)	59.4 (11.5)	< 0.01^6^
	Additional etiology of polyneuropathy	Etiology of polyneuropathy	
Diabetes mellitus, *n* (%)	1	21	
Metabolic^a^, *n* (%)	0	12	
Toxic^b^, *n* (%)	0	20	
Vitamin deficiency, *n* (%)	2	20	
Immune mediated^c^, *n* (%)	0	49	
Hereditary, *n* (%)	0	48	
Systemic disease^d^, *n* (%)	1	27	
Neuroborreliosis	0	1	
Idiopathic small fiber	-	20	
Multifactorial, *n* (%)	-	18	
Idiopathic axonal (CIAP), *n* (%)	-	92	

*CIAP*, chronic idiopathic axonal polyneuropathy

^a^Uremic, thyroid disorder

^b^Alcohol overuse, neurotoxic medication

^c^CIDP, Guillain Barre syndrome, IgM MGUS/anti-MAG related polyneuropathy, paraneoplastic, single organ vasculitis

^d^Vasculitis, amyloidosis, sarcoidosis, connective tissue disease

^e^Student’s *t*-test

^f^Fisher’s exact test

An additional etiology for polyneuropathy was present in 4 out of the 10 patients with ATTRv-PN. None of the patients with ATTRv-PN had a typical clinical presentation for CIAP: 3 had a family history of polyneuropathy and fast progression of symptoms; 1 patient had symptoms that were not length dependent and fasciculations in arms and legs early in the disease course; and the other 6 had fast progression (1 with walking disability within 5 years of symptom onset and 5 with decreased strength in the arms or upper legs at first presentation).

### Clinical phenotype

In total, 23 patients with ATTRv-PN were seen at our outpatient clinic, consisting of 10 patients from the yield of the genetic testing group outlined above, and an additional 13 patients who were referred with (suspected) ATTRv-PN. All patients had the pathogenic p.Val50Met variant. Table 2 shows the characteristics of the 15 patients with ATTRv-PN with no additional etiology for polyneuropathy. We did not include the 8 patients with ATTRv-PN and an additional etiology for polyneuropathy because this could have influenced the clinical phenotype (*n* = 1 alcohol abuse, *n* = 2 vitamin B12 deficiency, *n* = 1 AL-amyloidosis, *n* = 3 diabetes mellitus, *n* = 1 IgM MGUS/anti-MAG related polyneuropathy). Misdiagnosis occurred in 4 patients with ATTRv-PN; misdiagnosis included wild-type amyloidosis (*n* = 1), CIAP with benign fasciculation syndrome (*n* = 1), and CIDP (*n* = 2). The misdiagnosis of CIDP was based on ultrasound findings suggestive of inflammatory neuropathy with enlargements of the median nerve at the upper arm and/or forearm and brachial plexus and a progressive disease course. Nerve enlargement at pressure points was a common finding on nerve ultrasound (10/11 patients), and 3 patients had findings suggestive of inflammatory neuropathy. None of the patients with ATTRv-PN had motor nerve conduction study findings strongly supportive of demyelination [14].

**Table 2:** Comparison of clinical characteristics between patients with ATTRv-PN and patients with CIAP

	ATTRv-PN, *n* = 15	CIAP, *n* = 92	*P* value^a^
Male, *n* (%)	9 (60.0)	64 (72.8)	0.36
Age at TTR testing, mean (SD)	61.7 (10.2)	62.3 (9.8)	0.83
Age at symptom onset, mean (SD)	59.4 (11.1)	56.9 (15.3)	0.65
Symptom duration at TTR testing in years, median (IQR)	2.6 (4.5)	4 (5)	0.12^b^
Early onset (< 50 years)	3 (20.0)	25 (28.1)	0.75
Type of polyneuropathy			
Sensory	5 (33.3)	41 (44.6)	0.59
Sensorimotor	10 (66.7)	48 (55.4)	0.58
Pain dominant	8 (53.3)	39 (42.4)	0.58
Autonomic symptoms			
Erectile dysfunction, *n* (%)	7 (46.7)	19 (21.3)	0.05^b^
Sweating, *n* (%)	6 (40.0)	15 (16.9)	0.07
Defecation	6 (40.0)	8 (9.0)	0.01
Early satiation, *n* (%)	4 (26.7)	3 (3.4)	0.01^c^
Urinary symptoms, *n* (%)	6 (40.0)	21 (23.3)	0.21
Orthostatic hypotension, *n* (%)	6 (40.0)	20 (22.4)	0.20
Palpitations, *n* (%)	6 (40.0)	3 (3.4)	< 0.01^c^
Sicca, *n* (%)	2 (13.3)	16 (18.0)	1.0
Any, *n* (%)	13 (86.7)	54 (58.7)	0.01^4^
Number of autonomic symptoms (median, IQR)	3 (2.5)	1 (2)	< 0.01^b,c^
Decreased strength LL, *n* (%)	10 (66.7)	38 (41.3)	0.09
Decreased strength UL, *n* (%)	6 (40)	7 (7.6)	< 0.01^c^
CTS, *n* (%)	13 (86.7)	30 (38.5)	< 0.01^c^

*ATTRv-PN*, hereditary ATTR polyneuropathy; *CIAP*, chronic idiopathic axonal polyneuropathy; *IQR*, interquartile range; *LL*, lower limb; *UL*, upper limb; *CTS*, carpal tunnel syndrome

^a^Student’s *T* test for continuous outcomes, unless stated otherwise, and Fisher’s exact test for dichotomous outcomes

^b^Wilcoxon rank sum test with continuity correction

^c^*p* < 0.05 significant difference

^d^Fisher’s exact tests

Most patients with ATTRv-PN had a late-onset phenotype (> 50 years), and the age at symptom onset ranged from 39 to 71 and did not differ from patients with CIAP. CTS was more common in patients with ATTRv-PN and was mostly bilateral (12/13). Patients with ATTRv-PN more often had autonomic symptoms, and a higher number of autonomic symptoms, specifically erectile dysfunction, altered defecation pattern, early satiation, and palpitations, were seen more frequently compared to CIAP. Patients with ATTRv-PN more frequently had decreased strength in the arms. Type of polyneuropathy (sensory or sensorimotor; painful or not painful), progression rate, and location of symptoms, both at the onset of symptoms and at presentation, did not differ between patients with ATTRv-PN and those with CIAP.

Table 3 shows that all patients with ATTRv-PN would be identified by the TTR suspicion index. More than 70% of patients with CIAP who underwent testing also fulfilled the criteria for the TTR suspicion index. Most patients with ATTRv-PN had a family history of polyneuropathy, in 9 out of 11 in the first degree (ATTRv-PN: 60%, CIAP: 9%, p < 0.01), 2 patients had a family history of cardiomyopathy or sudden cardiac death and CTS, which can also be suggestive of ATTRv-PN. Patients with CIAP are not regularly assessed by an ophthalmologist; therefore, results for vitreous opacities were lacking. Symptom duration did not differ between CIAP and ATTRv-PN, also after the exclusion of patients who were tested because of a known mutation in their family (*n* = 8, median 5.9 years,* p* 0.25 using Fisher’s exact test).

**Table 3:** TTR suspicion index [1]

	ATTRv-PN, *n* = 15	CIAP, *n* = 92	*P* value^b^
Dysautonomia^a^, *n* (%)	13 (86.7)	47 (51.1)	0.01^c^
Early gait disorder, *n* (%)	4 (26.7)	4 (4.3)	< 0.01^c^
Weight loss > 5 kg, *n* (%)	2 (13.3)	5 (5.4)	0.25
Cardiac symptoms or history, *n* (%)	9 (60.0)	2 (2.2)	< 0.01^c^
Renal dysfunction, *n* (%)	0 (0)	0 (0)	1
Vitreous opacities, *n* (%)	3 (20.0)	NA	-
Bilateral CTS, *n* (%)	12 (80.0)	22 (23.9)	< 0.01^c^
Family history, *n* (%)	13 (86.7)	11 (12.0)	< 0.01^c^
Fulfilling TTR suspicion index (≥ 1 item fulfilled), *n* (%)	15 (100)	67 (72.8)	0.02^d^
≥ 2 items fulfilled, *n* (%)	14 (93.3)	21 (22.3)	< 0.01^c^

*CIAP*, chronic idiopathic axonal polyneuropathy; *ATTRv-PN*, hereditary ATTR polyneuropathy; *CTS*, carpal tunnel syndrome

^a^Altered defecation pattern, orthostatic hypotension, urinary symptoms, and erectile dysfunction

^b^Fisher’s exact test

^c^ < 0.05 significant differences

Patients with CIAP who underwent TTR gene sequencing more frequently had painful polyneuropathy (42% vs. 28%, *p* 0.03), CTS (39% vs. 23%, *p* 0.03), and autonomic symptoms (59% vs. 9%, *p* < 0.01), compared to patients with CIAP who were not tested. They also fulfilled the TTR suspicion index more often, although 60% of those who were not tested also fulfilled the suspicion index.

## Discussion

In this study, the yield of TTR gene sequencing in a population of patients with chronic polyneuropathy was 3% (10/338), but none of the identified patients with ATTRv-PN had a clinical presentation typical of CIAP. Therefore, routine testing for ATTRv-PN in patients with a typical presentation for CIAP does not appear to be useful. In all patients with ATTRv-PN, red flag symptoms were present at first presentation. Features that were more common in ATTRv-PN compared to CIAP included bilateral CTS, decreased strength in the upper limbs, autonomic symptoms, specifically altered defecation pattern, erectile dysfunction, early satiation and palpitations, cardiac involvement, family history suggestive of hATTRv, and having walking disability early in the disease course (i.e., needing a walking aid within 5 years of onset of polyneuropathy).

TTR mutations have previously been identified in patients with neuropathy without a definite etiology, although these patients also had atypical features [7]. Our findings are supported by 2 previous studies in which routine testing in patients with idiopathic neuropathy (including demyelinating and suspected hereditary polyneuropathy) and in patients with idiopathic small fiber or mixed neuropathy was not found to be useful [8, 9]. Our findings of distinguishing clinical features are supported by a German study that also described the early loss of mobility and involvement of the arms as clinical features which are suggestive of ATTRv-PN [11].

**Fig. 1:**
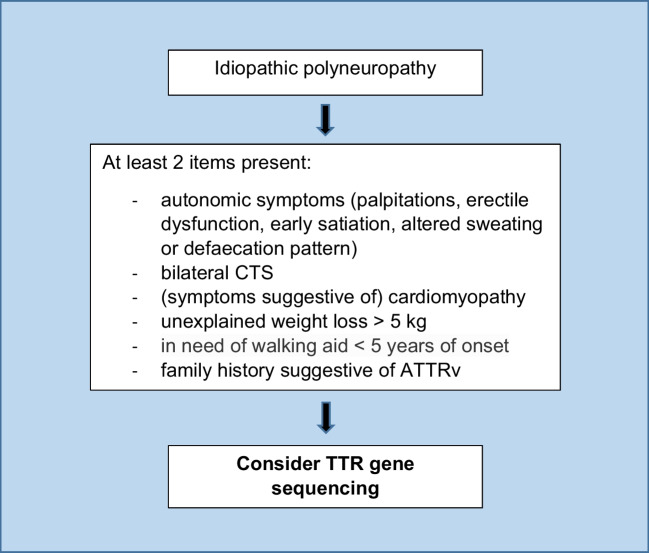
Revised TTR suspicion index

Over 70% of patients with CIAP fulfilled the suspicion index, and this was mostly due to the dysautonomia items that were present in a majority of patients. Orthostatic hypotension and urinary symptoms are indeed relatively common in an elderly population such as patients with CIAP and could be due to comorbidity or medication use. To achieve a higher diagnostic yield of genetic testing for TTR gene mutations, some alterations to the suspicion index might be appropriate. We suggest including the relatively uncommon symptoms of palpitations, erectile dysfunction, altered sweating and defaecation pattern, and early satiation in the dysautonomia items. The items renal dysfunction and vitreous opacities could be omitted because they did not have additional value and are not well applicable in a screening setting. Additionally, we suggest to require the presence of at least 2 items. With the combination of these alterations, all patients with ATTRv-PN are still identified, but the number of patients with CIAP who would require genetic testing is decreased from 67/92 (72.8%) using the original suspicion index to 17/92 (18%). Figure 1 shows our proposed revised version of the TTR suspicion index.

Misdiagnosis occurred in 4 patients with ATTRv-PN; misdiagnosis included wild-type amyloidosis (*n* = 1), CIAP with benign fasciculation syndrome (*n* = 1), and CIDP (*n* = 2). The misdiagnosis of CIDP was based on ultrasound findings suggestive of inflammatory neuropathy with enlargements of the median nerve at the upper arm and/or forearm and brachial plexus and a progressive disease course. Nerve enlargement at pressure points was a common finding on nerve ultrasound (10/11 patients), and 3 patients had findings suggestive of inflammatory neuropathy. None of the patients with ATTRv-PN had motor nerve conduction study findings strongly supportive of demyelination.

The strengths of our study are the stringent criteria used for the diagnosis of polyneuropathy and CIAP. Because we excluded patients with ATTRv-PN who had an additional etiology of polyneuropathy, there is a lower risk of confounding of the clinical phenotype. Due to the retrospective nature of the study, there is a risk of information bias, as it is possible that certain symptoms, such as autonomic symptoms and cardiac symptoms and family history, were more frequently reported and more elaborately asked about in patients with ATTRv-PN. Because of the rarity of ATTRv in the Netherlands, the number of included patients with ATTRv-PN was small and this limited our possibilities in statistical analysis. In our cohort, we only have patients with the p.Val50Met mutation; therefore, our results might not be applicable to patients with other pathogenic mutations in the TTR gene. There was selection bias in the patients with CIAP who underwent genetic testing. This was to be expected as genetic testing was carried out based on (low) clinical suspicion; as a result, patients with neuropathic pain, autonomic symptoms, and faster progression rate were tested more often. The group of patients with CIAP who did not undergo genetic testing might still contain patients with ATTRv-PN such as very late-onset cases without a family history, but this seems highly unlikely in the absence of suspect clinical features as none of the identified patients with ATTRv-PN in this study had a typical presentation for CIAP. However, because of these limitations, it is important that our suggested revised TTR suspicion index is validated.

In conclusion, carrying out routine genetic testing in patients with a typical clinical presentation for CIAP does not appear to be useful. A thorough patient and family history, including CTS and sudden cardiac death/cardiomyopathy, is paramount to detect red flag symptoms for ATTRv-PN. The suspicion index can be used to select patients in whom testing is useful, but we suggest a revised version to increase diagnostic yield.

- **What is already known on this topic** – *Diagnostic delay and misdiagnosis occur frequently in patients with ATTRv-PN due to a variable phenotype, but should be prevented since treatments have become available that are most effective early in the disease. It is not known whether misdiagnosis also occurs in patients diagnosed with CIAP. It is also not known which clinical features can differentiate between patients with ATTRv-PN and those with CIAP in a non-endemic area.*
- **What this study adds** – *This study shows that it is not useful to routinely test for TTR mutations in patients with a typical clinical presentation for CIAP; however, some clinical features are suggestive of ATTRv-PN.*
- **How this study might affect research, practice, or policy** – *Routine testing for ATTRv-PN can be omitted in patients with a presentation typical for CIAP; this study provides guidance for the selection of patients in whom testing should be considered.*

## Supplementary Information

Below is the link to the electronic supplementary material.

Supplementary file1 (DOCX 31 KB)

## Data Availability

Data are available on reasonable request.
