# Supplementary material for: Red flags and adjusted suspicion index for distinguishing hereditary transthyretin amyloid polyneuropathy from idiopathic axonal polyneuropathy
Source: Neurol Sci. 2023 Jun 2;44(10):3679–85. doi: 10.1007/s10072-023-06859-w (PMC10495272; doi:10.1007/s10072-023-06859-w)
Supplement: Supplementary file 1 — Supplementary file1 (DOCX 31 KB) [file 10072_2023_6859_MOESM1_ESM.docx]

**Supplementary data**

Red flags and adjusted suspicion index for distinguishing hereditary transthyretin amyloid polyneuropathy from idiopathic axonal polyneuropathy

| **Table 4**  Comparison of clinical characteristics between patients with CIAP who underwent genetic testing and those who did not. | | | | |
| --- | --- | --- | --- | --- |
|  | CIAP, n = 92 | CIAP not tested, n=162 | P value^a^ |  |
| Male n (%) | 64 (72.8) | 105 (64.8) | 0.24 |  |
| Age at TTR testing, mean (SD) | 62.3 (9.8) | - | - |  |
| Age at symptom onset, mean (SD) | 56.9 (15.3) | 59.0 (9.9) | 0.11 |  |
| Symptom duration at TTR testing in years, median (IQR) | 4 (5) | - | - |  |
| Early onset (<50 yrs.) | 25 (28.1) | 29 (18.1) | 0.10 |  |
| Type of polyneuropathy |  |  |  |  |
| Sensory | 41 (44.6) | 75 (46.3) | 0.89 |  |
| Sensorimotor | 48 (55.4) | 87 (53.7) | 0.89 |  |
| Pain dominant | 39 (42.4) | 45 (27.8) | 0.03^b^ |  |
| Autonomic symptoms |  |  |  |  |
| Erectile dysfunction, n (%) | 19 (21.3) | 13 (9.6) | 0.02^b^ |  |
| Sweating, n (%) | 15 (16.9) | 4 (3.0) | <0.01^b^ |  |
| Defecation | 8 (9.0) | 6 (4.4) | 0.27 |  |
| Early satiation, n (%) | 3 (3.4) | 0 (0) | 0.06^c^ |  |
| Urinary symptoms, n (%) | 21 (23.3) | 10 (7.4) | <0.01^b^ |  |
| Orthostatic hypotension, n (%) | 20 (22.4) | 13 (9.6) | 0.01^b^ |  |
| Palpitations, n (%) | 3 (3.4) | 1 (0.7) | 0.30^c^ |  |
| Sicca, n (%) | 16 (18.0) | 11 (8.1) | 0.05 |  |
| Any, n (%) | 54 (58.7) | 35 (9.4) | <0.01^b^ |  |
| Number of autonomic symptoms (median, IQR) | 1 (2) | 0 (1) | <0.01^b^ |  |
| Decreased strength LL, n (%) | 38 (41.3) | 53 (60.3) | 0.41 |  |
| Decreased strength UL, n (%) | 7 (7.6) | 7 (4.3) | 0.96 |  |
| CTS, n (%) | 30 (38.5) | 26 (22.8) | 0.03^b^ |  |

Abbreviations: CIAP = chronic idiopathic axonal polyneuropathy, IQR = interquartile range, LL = lower limb, UL = upper limb, CTS= carpal tunnel syndrome

Legend:

^a^ Students T test for continuous variables, unless stated otherwise, and χ2 test for dichotomous outcomes

^b^ p <0.05 significant difference

^e^ Fisher exact test

| Table 5  TTR Suspicion Index [1] | | | | |
| --- | --- | --- | --- | --- |
|  | CIAP n = 92 | CIAP not tested, n=162 | P value^b^ |  |
| Dysautonomia^a^, n (%) | 47 (51.1) | 31 (19.1) | <0.01^c^ |  |
| Early gait disorder, n (%) | 4 (4.3) | 5 (3.1) | 0.73 |  |
| Weight loss >5kg, n (%) | 5 (5.4) | 6 (3.7) | 0.53 |  |
| Cardiac symptoms or history, n (%) | 2 (2.2) | 2 (1.2) | 0.62 |  |
| Renal dysfunction, n (%) | 0 (0) | 4 (2.5) | 1 |  |
| Vitreous opacities, n (%) | NA | NA | - |  |
| Bilateral CTS, n (%) | 22 (23.9) | 15 (9.4) | 0.03^c^ |  |
| Family history, n (%) | 11 (12.0) | 16 (9.9) | 0.88 |  |
| Fulfilling TTR Suspicion Index (≥1 item fulfilled), n (%) | 67 (72.8) | 66 (59.3) | <0.01^c^ |  |
| ≥2 items fulfilled, n (%) | 21 (22.3) | 7 (2.9) | 0.01^c^ |  |

Abbreviations: CIAP = chronic idiopathic axonal polyneuropathy, CTS = carpal tunnel syndrome

Legend:

^a^ altered defecation pattern, orthostatic hypotension, urinary symptoms and erectile dysfunction

^b^ χ2 test

^c^ <0.05 significant difference

| Table 6 Fulfilment of suspicion index items in patients with ATTRv-PN | | | | | | | | | |
| --- | --- | --- | --- | --- | --- | --- | --- | --- | --- |
| Patient | Dysautonomia^a^ | Gait disorder | Weight loss >5 kilo | Cardiac symptoms/history | Renal dysfunction | Vitreous opacities | Bilateral CTS | Family history^b^ | Number of items present |
| 1 | √ | √ | - | √ | - | - | √ | √ (first degree) | 5 |
| 2 | - | - | - | - | - | - | - | √^c^ | 1 |
| 3 | √ | - | - | - | - | NA | - | √^c^ | 2 |
| 4 | √ | √ | - | - | - | NA | √ | - | 3 |
| 5 | √ | - | - | √ | - | NA | √ | - | 3 |
| 6 | √ | - | - | - | - | - | √ | √^c^ | 3 |
| 7 | √ | - | - | - | - | √ | √ | √^c^ | 4 |
| 8 | √ | - | - | - | - | √ | √ | √^c^ | 4 |
| 9 | √ | - | - | √ | - | - | √ | √^c^ | 4 |
| 10 | √ | - | - | √ | - | NA | - | √^c^ | 3 |
| 11 | √ | √ | √ | √ | - | - | √ | - | 5 |
| 12 | √ | √ | - | √ | - | - | √ | √ | 5 |
| 13 | √ | √ | √ | √ | - | √ | √ | √ (first degree) | 7 |
| 14 | √ | - | - | √ | - | - | √ | √ (first degree) | 4 |
| 15 | - | - | - | √ | - | - | √ | √ | 3 |

Abbreviations: ATTRv-PN = hereditary ATTR polyneuropathy, CTS = carpal tunnel syndrome

Legend:

^a^ Altered defecation pattern, orthostatic hypotension, urinary symptoms and erectile dysfunction

^b^ Family history (suggestive of) ATTRv, including: known TTR mutation, polyneuropathy, cardiomyopathy, arrhythmia, sudden heart death, and/or CTS

^c^ Known TTR mutation in family

| Table 7 EAN/PNS 2021 electrodiagnostic criteria supportive of CIDP applied to patients with ATTRv-PN | | | | | | | | | |
| --- | --- | --- | --- | --- | --- | --- | --- | --- | --- |
| Patient | DML ↑  (in 2 nerves) | MCV ↓  (in 2 nerves) | F-wave ↑  (in 2 nerves) | F-wave absent  (in 2 nerves  + 1 other item in 1 other nerve) | Conduction block  (in 2 nerves) | Abnormal temporal dispersion  (in 2 nerves) | CMAP duration ↑  (+1 other item in 1 other nerve) | Motor nerve conduction criteria fulfilled | Ultrasound criteria fulfilled |
| 1 | - | - | - | - | - | - | - | No | NA |
| 2 | - | - | - | - | - | - | - | No | NA |
| 3 | - | - | - | 1 nerve^a^ | - | - | - | No | NA |
| 4 | - | - | 1 nerve^a^ | - | - | - | - | Weakly supportive | NA |
| 5 | - | - | - | 1 nerve^a^ | - | - | - | No | No |
| 6 | - | - | - | - | - | - | - | No | No |
| 7 | - | - | - | - | - | - | - | No | No |
| 8 | - | - | - | 1 nerve^c^ | - | - | - | No | No |
| 9 | - | - | - | - |  | - | - | No | Yes |
| 10 | - | - | - | - | - | - | 1 nerve^a^ | No | Yes |
| 11 | - | - | - | - | - | - | 2 nerves^a,b^ | No | No |
| 12 | - | - | - | - | - | - | - | No | No |
| 13 | - | - | - | - | - | - | - | No | No |
| 14 | - | - | - | - | - | - | - | No | Yes |
| 15 | - | - | 1 nerve^b^ | - | - | - | - | Weakly supportive | No |

Abbreviations: DML = distal motor latency, MCV = motor conduction velocity, CMAP = compound muscle action potential, NA = not available

Legend: ^a^ Median nerve

^b^ Ulnar nerve

^c^ Peroneal nerve
